# Identifying Predictors of Childhood Anaemia in North-East India

**DOI:** 10.3329/jhpn.v31i4.20001

**Published:** 2013-12

**Authors:** Sanku Dey, Sankar Goswami, Tanujit Dey

**Affiliations:** ^1^Department of Statistics, St. Anthony's College, Shillong, India; ^2^Department of Statistics, G.C. College, Silchar, India; ^3^Department of Mathematics, The College of William and Mary, Williamsburg, VA, USA

**Keywords:** Anaemia, Multiple logistic regression, Odds ratio, Wald Test statistic, India

## Abstract

The objective of this study is to examine the factors that influence the occurrence of childhood anaemia in North-East India by exploring dataset of the Reproductive and Child Health-II Survey (RCH-II). The study population consisted of 10,137 children in the age-group of 0-6 year(s) from North-East India to explore the predictors of childhood anaemia by means of different background characteristics, such as place of residence, religion, household standard of living, literacy of mother, total children ever born to a mother, age of mother at marriage. Prevalence of anaemia among children was taken as a polytomous variable. The predicted probabilities of anaemia were established via multinomial logistic regression model. These probabilities provided the degree of assessment of the contribution of predictors in the prevalence of childhood anaemia. The mean haemoglobin concentration in children aged 0-6 year(s) was found to be 11.85 g/dL, with a standard deviation of 5.61 g/dL. The multiple logistic regression analysis showed that rural children were at greater risk of severe (OR=2.035; p=0.003) and moderate (OR=1.23; p=0.003) anaemia. All types of anaemia (severe, moderate, and mild) were more prevalent among Hindu children (OR=2.971; p=0.000), (OR=1.195; p=0.010), and (OR=1.201; p=0.011) than among children of other religions whereas moderate (OR=1.406; p=0.001) and mild (OR=1.857; p=0.000) anaemia were more prevalent among Muslim children. The fecundity of the mother was found to have significant effect on anaemia. Women with multiple children were prone to greater risk of anaemia. The multiple logistic regression analysis also confirmed that children of literate mothers were comparatively at lesser risk of severe anaemia. Mother's age at marriage had a significant effect on anaemia of their children as well.

## INTRODUCTION

Childhood anaemia is a major public-health concern, with an increasing risk of mortality. Ministry of Health and Family Welfare, Government of India, reports that it is one of the most common diseases due to nutritional deficiency in the world today, and more than half of the population in India is anaemic. The prevalence of anaemia is as high as 70–80% among children and 60% among pregnant women (1). Anaemia is a common conundrum of nutritional deficiency worldwide, and its prevalence is higher in developing countries than developed countries (2,3).

Almost 34% of the world population suffers from iron deficiency, with 80% belonging to developing countries where the prevalence of anaemia and iron deficiency is approximately 40% whereas, in developed countries, the occurrence of anaemia is lower than 10% (4). WHO lists iron-deficiency anaemia (IDA) as one of the “top ten risk factors contributing to death” (5). IDA is prevalent in South Asia, predominantly in India, Bangladesh, and Pakistan. However, the prevalence of IDA in Bangladesh and Pakistan has declined to 55% (6). The waning prevalence of IDA is remarkable in the case of China where the occurrence rate has plummeted from 20% to 8% within a decade (7). It is difficult to ascertain the true incidence of IDA as the aetiology of anaemia is multifactorial. A large-scale study conducted by Indian Council of Medical Research (ICMR) found that about 53% of children were anaemic (8). A study by National Family Health Survey-2 (NFHS-2) (9) found the occurrence of anaemia among children aged 1–5 year(s) a little lower than adolescents and women of childbearing age, who are at risk of developing anaemia (10). IDA is a public-health crisis in India, especially among pregnant and lactating women, children, and adolescents (11). Over the last 50 years, the prevalence of iron-deficiency anaemia has varied between 68 and 97% among children (12,13). Different studies in India (14), Indonesia (15), Thailand (16), and the United States (17) have shown that iron-deficiency anaemia leads to psychomotor retardation, low intelligence, and decreased learning capability in children below 5 years and primary school students. The different forms of anaemia also endanger livelihood (5,18).

A number of studies have been conducted on the association between the socioeconomic status (SES) and the prevalence of anaemia (19-21). As SES is a significant determinant of access to healthcare, a large number of people live with no or restricted access to medical attention and preventive measures (22), which results in ever-increasing risk of becoming anaemic.

The factors that influence the occurrence of anaemia in a population are fundamental to the implementation of control measures. Bearing this in mind, our aim was to determine the prevalence of anaemia among children aged 0-6 year(s) from the northeastern states of India and to identify the predictors that are significantly associated with anaemia. To comprehend the prevalence of anaemia, socioeconomic differentials have also been taken into consideration.

## MATERIALS AND METHODS

### Data

The study used data from Reproductive and Child Health-II Survey, 2002-2004 (23) for the northeastern states of India. The RCH-II survey was carried out throughout India in two phases by the Ministry of Health and Family Welfare, Government of India and funded by the World Bank. International Institute for Population Sciences (IIPS), Mumbai, collected the data as a nodal agency with the help of different regional agencies in various districts of the country. The survey used a systematic multistage stratified sampling; the stages of selection were districts, primary sampling units (PSUs), and households; 1,000 representative households were identified for the survey, using appropriate sampling procedure from each district. Thirty percent of the sample was selected from urban areas and was based on National Sample Survey Organisation (NSSO) urban sampling frame. The survey provided district-level information on the prevalence of undernutrition [weight-for-age, using the standard deviation (SD) classification] among children in the age-group of 0-72 month(s); prevalence of anaemia (Hb estimation by indirect cyanmethemoglobin method) in children aged 0-72 month(s), adolescent girls aged 10-19 year(s), and pregnant women; household availability of iodized salt; and the coverage of vitamin A programme, with appropriate dosage. Details of the survey are available elsewhere (23). To meet the objectives of the study, we produced a file that pertains to the northeastern states of India. Our study comprises 10,137 children within 0-6 year(s) of age.

Levels of anaemia were classified as severe, moderate, and mild, based on the haemoglobin concentrations and according to the specification of the World Health Organization (24). Severe anaemia was diagnosed as haemoglobin concentration less than 7.0 g/dL, moderate anaemia as haemoglobin concentration 7.0-9.9 g/dL, and mild anaemia as haemoglobin concentration 10.0-10.9 g/dL.

### Method of analysis

In this study, a predictive model on childhood anaemia was developed by using multinomial logistic regression technique. To assess the degree of association between the risk factors and anaemia, odds ratios were computed. The data on predictors were based on: place of residence, standard of living, sex of the child, literacy of mother, total number of children ever born to a mother, and age of the mother at marriage. The response variable was designed as polytomous anaemia level Y (1=severely anaemic, 2=moderately anaemic, 3=mildly anaemic, and 4=non-anaemic).

Wald Test statistic was used in testing the significance of the logistic regression coefficients. SPSS (version 11.0) was used for analyzing the data, and the last category of each predictor was taken as the reference group.

The multinomial logistic regression model is an extension of the binary logistic regression model where the dependent variable is polytomous, i.e. its values consist of more than two categories. In such cases, if we assume that the possible numbers of categories are q, then we need q-1 logits. The logit multinomial model can be written as:

(1)
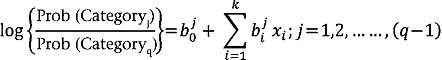


where 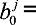 intercept for the j-th logit, 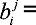 regression coefficient for *i*-th predictor *x_i_* in the *j*-th logit, *k*=number of predictors in the model.

In the above expression, one of the categories is used as reference and is called the baseline category. In our study, the different categories are the different levels of anaemia. As a result, we consider non-anaemic as the reference category. After estimating the coefficients of the model (Equation 1) via the method of maximum likelihood, we were able to compute the logits and, hence, the probabilities of each of the categories. The equations for the multiple logistic regressions are:

(2)
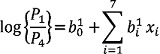


(3)
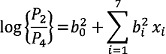


(4)
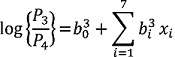


where *P*_1_=Probability of getting severely anaemic,

*P*_2_=Probability of getting moderately anaemic,

*P*_3_=Probability of getting mildly anaemic,

*P*_4_=Probability of getiing non-anaemic,

and *P*_1_+ *P*_2_+*P*_3_=1; {*x*_i_}'s (i=1,2,…,7)

are the aforesaid predictors.; 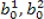 and 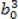 are the regression coefficients of Equation 2 to 4. Table 1 through 3 represent an overview of the predictors used in the model as well as the sociodemographic temperament of the population.

## RESULTS

In this study, the mean haemoglobin concentration of children of age-group 0-6 year(s) in North-East India was found to be 11.85 g/dL, with a standard deviation of 5.61 g/dL. Sociodemographic characteristics of anaemic and non-anaemic groups are presented in Table 3. The data show that, out of 10,137 children in North-East India, 52.5% were anaemic (1.9% severely anaemic, 24.7% moderately anaemic, and 25.9% mildly anaemic). Table 2 shows that the highest proportion of anaemic children were found in Tripura (74.2%), followed by Sikkim (69.9%) and Assam (61.8%). It was also observed that the prevalence of anaemia was 53.1% and 51.9% among male and female children respectively.

The study reveals that rural children were at greater risk of severe (OR=2.035; p=0.003) and moderate (OR=1.230; p=0.003) anaemia compared to urban children (Table 4.1 and 4.2). Data show that male children were at greater risk of having severe anaemia than female children (O.R=1.488; p=0.010) (Table 4.1). Our analysis suggests that children born to literate women were more likely to have moderate (OR=1.126; p=0.036) and mild (OR=1.047; p=0.412) anaemia compared to children born to non-literate women (Table 4.2). Results show that all types of anaemia (severe, moderate, and mild) were more prevalent (OR=2.971; p=0.000), (OR=1.195; p=0.010), and (OR=1.201; p=0.011) among Hindu children than children of other religious groups whereas moderate (OR=1.406; p=0.001) and mild (OR=1.857; p=0.000) anaemia were more prevalent among Muslim children (Table 4.1, 4.2, and 4.3). This indicates that, compared to children of other communities, the associated risks of anaemia for Hindu children will increase by 2.971 for severe, 1.195 for moderate, and 1.201 for mild aneamia. The said risks for Muslim children will increase by 1.748 times for severe, 1.406 times for moderate, and 1.857 times for mild anaemia.

**Table 1.** Description of predictors in the logistic regression model

Predictor	Name of variable and value level	Type of variable
X1	Place of residence	Nominal
	1=Rural, 2=Urban	Categorical
X2	Religion	
	1=Hindu, 2=Muslim	Nominal
	3=Christian, 4=Others	Categorical
X3	Household standard of living	Ordinal
	1=Low, 2=Medium, 3=High	Categorical
X4	Sex of the child	Nominal
	1=Male, 2=Female	Categorical
X5	Literacy of mother	
	1=Can read and write	Ordinal
	2=Can't read and write	Categorical
X6	Total no. of children ever born to a	
	mother 1=Up to two, 2=Three or four	Nominal
	3=Five or above	Categorical
X7	Age of mother at marriage	
	1=Below 18 years, 2=18-26 years	Nominal
	3=Above 26 years	Categorical

**Table 2.** State vs anaemia level cross-tabulation

State	Anaemia level				Total
	Severely anaemic	Moderately anaemic	Mildly anaemic	Non-anaemic	
Sikkim	29 4.1%	283 40.0%	190 26.8%	206 29.1%	708 100.0%
Arunachal Pradesh	24 0.8%	639 21.9%	639 21.9%	1,611 55.3%	2,913 100.0%
Nagaland	6 1.1%	82 15.6%	160 30.4%	278 52.9%	526 100.0%
Manipur	17 0.8%	457 21.9%	411 19.7%	1198 57.5%	2,083 100.0%
Mizoram	13 2.0%	138 20.8%	215 32.5%	296 44.7%	662 100.0%
Tripura	6 1.7%	156 43.2%	106 29.4%	93 25.8%	361 100.0%
Meghalaya	6 3.0%	34 17.1%	54 27.1%	105 52.8%	199 100.0%
Assam	94 3.5%	710 26.4%	855 31.8%	1026 38.2%	2,685 100.0%
Total	195 1.9%	2,499 24.7%	2,630 25.9%	4,813 47.5%	10,137 100.0%

Numbers represent cell frequencies and their corresponding proportions

The odds ratios and p values in both severe and moderate groups suggest that children from households with low and medium standard of living were more likely to have anaemia (OR=1.306; p=0.381; OR=1.609; p=0.089) and (OR=1.165; p=0.102; OR=1.164; p= 0.103) compared to those with high standard of living (Table 4.1 and 4.2).

Mother's fertility also appeared to be closely associated with anaemia in their children. Results show that the risk of anaemia increased with the fertility of mother, i.e. more the number of children ever born to a mother, the more the possibility of the children being anaemic. The odds ratios also reflect that higher the mother's age at marriage, the more the likelihood of the children being mildly anaemic (Table 4.1, 4.2, and 4.3).

Results illustrate that certain factors, namely place of residence, religion, household standard of living, gender of the child, and total number of children ever born to a mother, stimulate anaemia in children (Table 5).

## DISCUSSION

In this study, high prevalence of anaemia was observed among children aged 0-6 year(s) in the northeastern states of India. This trend is widespread not only in India and Brazil but also in other developing countries. In New Zealand, the prevalence of anaemia was found to be 49% among children aged 6-11 months and 22% among children aged 12-24 months (25). In Viet Nam, 45.1% of children below the age of 5 years were found to be suffering from anaemia (26). In sub-Saharan African countries, prevalence of anaemia was recorded to be 82% in Benin and 83% in Mali (27).

**Table 3.** Sociodemographic characteristics of the two groups (N=10,137)

Variable	Severely anaemic	Moderately anaemic	Mildly anaemic	Non-anaemic	Total
Place of residence					
Urban	171 (2.1%)	2,052 (25.4%)	2,033(25.2%)	3,818 (47.3%)	8,074 (100%)
Rural	24 (1.2%)	447 (21.7%)	597 (28.9%)	995 (48.2%)	2,063 (100%)
Religion					
Hindu	116 (3.1%)	1,024 (27.1%)	965 (25.6%)	1,667 (44.2%)	3,772 (100%)
Muslim	14 (1.5%)	250 (27.1%)	309 (33.5%)	350 (37.9%)	923 (100%)
Christian	40 (1.2%)	713 (20.5%)	894 (25.7%)	1,825 (52.6%)	3,472 (100%)
Others	25 (1.3%)	512 (26.0%)	462 (23.5%)	971 (49.3%)	1,970 (100%)
Household standard of living					
Low	116 (1.9%)	1,544 (25.1%)	1,535 (25.0%)	2,956 (48.1%)	6,151(100%)
Medium	64 (2.2%)	695 (24.4%)	775 (27.2%)	1,312 (46.1%)	2,846 (100%)
High	15 (1.3%)	260 (22.8%)	320 (28.1%)	545 (47.8%)	1,140 (100%)
Sex of the child					
Male	117 (2.2%)	1,294 (24.7%)	1,365 (26.1%)	2,453 (46.9%)	5,229 (100%)
Female	78 (1.6%)	1,205 (24.6%)	1,265 (25.8%)	2,360 (48.1%)	4,908 (100%)
Literacy of mother					
Can read and write	105 (1.8%)	1,464 (24.9%)	1,532 (26.1%)	2,772 (47.2%)	5,873 (100%)
Can't read and write	78 (2.0%)	959 (24.6%)	993 (25.5%)	1,867 (47.9%)	3,897 (100%)
Total no. of children ever born to a mother					
Up to two	91 (2.1%)	1,115 (26.0%)	1,062 (24.8%)	2,015 (47.0%)	4,283 (100%)
Three or four	59 (1.6%)	885 (23.8%)	964 (26.0%)	1,805 (48.6%)	3,713 (100%)
Five or above	33 (1.9%)	23 (23.8%)	499 (28.1%)	819 (46.2%)	1,774 (100%)
Age of mother at marriage					
Below 18 years	54 (1.7%)	771 (24.6%)	758 (24.1%)	1,556 (49.6%)	3,139 (100%)
18-26 years	121 (2.0%)	1,532 (25.0%)	1,630 (26.6%)	2,847 (46.4%)	6,130 (100%)
Above 26 years	8 (1.6%)	120 (24.0%)	137 (27.3%)	236 (47.1%)	501 (100%)

Numbers represent cell frequencies and their corresponding proportions

According to National Family Health Survey-3 (NFHS-3), 79% of chil­dren aged 6-59 months were anaemic, including 40% who were moderately anaemic and 3% who were severely anaemic. The only states where less than half of the children were anaemic are Goa (38%), Manipur (41%), Mizoram (44%), and Kerala (45%). In contrast to the findings of NFHS-3, our study reveals that, in Arunachal Pradesh (44.7%), Nagaland (47.1%), Manipur (42.5%), and Meghalaya (47.2%), less than half of the children were anaemic. In conformity to our finding, several other studies (28-31) carried out on childhood anaemia indicated that children living in rural areas were at greater risk of anaemia compared to their urban counterparts. While some studies (28,32) reported that there was no association between gender and anaemia, others as well as our results showed that male children were at greater risk of anaemia than female children (29,33).

Children in households with low and medium standard of living index were more likely to have anaemia compared to their counterparts, which corroborates the findings of studies carried out in Brazil and other countries (34,35). The literacy factor shows that children of literate mothers were comparatively at lesser risk of severe anaemia than children of non-literate mothers but they too were at higher risk of moderate and mild anaemia. This confirms the findings of National Family Health Survey-3 (1), which revealed that more than half of the children were anaemic even when their mothers had 12 or more years of schooling or were in the highest wealth quintile. The fertility of mother was found to have significant effect on anaemia. Multiple children in the family increased the risk of anaemia enormously. Greater the number of children in the household, the greater the needs of the family in terms of domestic work, care of the children, and demand for food, which might possibly heighten the risk of anaemia (36,37). Mother's age at marriage had a considerable effect on anaemia in their children. Children of women who got married between 18 and 26 years were at greater risk of anaemia (33,38). The outcome of our study suggests that socioeconomic factors also influence childhood anaemia. In our study, we have used haemoglobin concentration as a proxy indicator of iron deficiency. Other indicators, such as serum ferritin (39), have not been delved into. Dietary intake, an important determinant of iron deficiency, together with worm infestations, malaria, and the role of infectious diseases, have also been excluded from the study.

**Table 4.1:** Parameters of multiple logistic regression model (Group: Severe anaemia)

Predictor	*β^ˆ^*	SE (*β^ˆ^*)	Wald test	df	p value	Odds ratio	95% CI for OR	
							Lower	Upper
Intercept	-4.649	556	69.927	1	0.000	-	-	-
[X1=1]	0.710	0.239	8.860	1	0.003	2.035	0.275	3.248
[X1=2]	0	-	-	0	-	-	-	-
[X2=1]	1.089	0.233	21.914	1	0.000	2.971	1.883	4.688
[X2=2]	0.558	0.353	2.496	1	0.114	1.748	0.874	3.494
[X2=3]	-9.417E-02	0.270	0.122	1	0.727	0.910	0.537	1.544
[X2=4]	0	-	-	0	-	-	-	-
[X3=1]	0.267	0.305	0.766	1	0.381	1.306	0.718	2.377
[X3=2]	0.512	0.312	2.897	1	0.089	1.669	0.925	3.009
[X3=3]	0	-	-	0	-	-	-	-
[X4=1]	0.398	0.155	6.596	1	0.010	1.488	1.099	2.016
[X4=2]	0	-	-	0	-	-	-	-
[X5=1	-6.661E-02	0.169	0.154	1	0.694	0.936	0.671	1.304
[X5=2]	0	-	-	0	-	-	-	-
[X6=1]	-0.101	0.222	0.209	1	0.648	0.904	0.585	1.396
[X6=2]	-0.287	0.226	1.612	1	0.204	0.751	0.482	1.169
[X6=3]	0	-	-	0	-	-	-	-
[X7=1]	-0.251	0.394	0.407	1	0.523	0.778	0.360	1.683
[X7=2]	5.835E-02	0.375	0.024	1	0.876	1.060	0.509	2.209
[X7=3]	0	-	-	0	-	-	-	-

**Table 4.2:** Parameters of multiple logistic regression model (Group: Moderate anaemia)

Predictor	β	SE (β)	Wald test	df	p value	Odds ratio	95% CI for OR	
							Lower	Upper
Intercept	0.908	0.168	29.327	1	0.000	-	-	-
[X1=1]	0.207	0.070	8.595	1	0.003	1.230	1.071	1.412
[X1=2]	0	-	-	0	-	-	-	-
[X2=1]	0.178	0.069	6.591	1	0.010	1.195	1.043	1.369
[X2=2]	0.341	0.103	10.901	1	0.001	1.406	1.148	1.721
[X2=3]	-0.0339	0.074	21.126	1	0.000	0.713	0.617	0.824
[X2=4]	0	-	-	0	-	-	-	-
[X3=1]	0.153	0.094	2.675	1	0.102	1.165	0.970	1.400
[X3=2]	0.152	0.093	2.661	1	0.103	1.164	0.970	1.398
[X3=3]	0	-	-	0	-	-	-	-
[X4=1]	2.952E-02	0.050	0.342	1	0.559	1.030	0.933	1.137
[X4=2]	0	-	-	0	-	-	-	-
[X5=1]	0.119	0.057	4.383	1	0.036	1.126	1.008	1.259
[X5=2]	0	-	-	0	-	-	-	-
[X6=1]	-1.058E-02	0.075	0.020	1	0.888	0.989	0.854	1.146
[X6=2]	-6.968E-02	0.074	0.883	1	0.347	0.933	0.807	1.079
[X6=3]	0	-	-	0	-	-	-	-
[X7=1]	-0.189	0.124	2.339	1	0.126	0.828	0.650	1.055
[X7=2]	-3.293E-02	0.118	0.078	1	0.780	0.968	0.768	1.219
[X7=3]	0	-	-	0	-	-	-	-

**Table 4.3:** Parameters of multiple logistic regression model (Group: Mild anaemia)

Predictor	*β^ˆ^*	SE (*β^ˆ^*)	Wald test	df	p value	Odds ratio	95% CI for OR	
							Lower	Upper
Intercept	0.386	0.161	5.774	1	0.016	-	-	-
[X1=1]	-7.202E-02	0.066	1.188	1	0.276	0.931	0.817	1.059
[X1=2]	0	-	-	0	-	-	-	-
[X2=1]	0.183	0.072	6.529	1	0.011	1.201	1.043	1.381
[X2=2]	0.619	0.101	37.709	1	0.000	1.857	1.524	2.262
[X2=3]	-2.036E-02	0.073	0.078	1	0.781	0.980	0.849	1.131
[X2=4]	0	-	-	0	-	-	-	-
[X3=1]	-5.994E-02	-0.090	0.448	1	0.503	0.942	0.790	1.122
[X3=2]	7.208E-02	0.088	0.670	1	0.413	1.075	0.904	1.277
[X3=3]	0	-	-	0	-	-	-	-
[X4=1]	3.380E-02	0.050	0.435	1	0.510	1.033	0.937	1.139
[X4=2]	0	-	-	-	-	-	-	-
[X5=1]	4.623E-02	0.056	0.674	1	0.412	1.047	0.938	1.170
[X5=2]	0	-	-	0	-	-	-	-
[X6=1]	-0.232	0.073	10.154	1	0.001	0.793	0.688	0.915
[X6=2]	-0.160	0.071	5.095	1	0.024	0.852	0.741	0.979
[X6=3]	0	-	-	0	-	-	-	-
[X7=1]	-0.306	0.119	6.594	1	0.010	0.736	0.583	0.930
[X7=2]	-7.728E-02	0.113	0.470	1	0.493	0.926	0.742	1.154
[X7=3]	0	-	-	0	-	-	-	-

**Table 5.** Likelihood ratio test

Effect	-2 Log likelihood of reduced model	Chi-square	df	Significance
Intercept	4169.141	0 .000	0	-
X1	4192.333	23.192	3	0.000
X2	4322.554	153.413	9	0.000
X3	4180.928	11.787	6	0.067
X4	4176.027	6.887	3	0.076
X5	4173.977	4.836	3	0.184
X6	4183.501	14.361	6	0.026
X7	4191.094	21.953	6	0.001

df=Degree of freedom

### Conclusions

This paper evaluated the existing endemicity of childhood anaemia in northeastern states of India and summarized the available information to present the extent of occurrence of anaemia among children aged 0-6 year(s). The results suggest that the place of residence, religion, gender of the child, household standard of living, and total number of children ever born to a mother impacted the risk of anaemia in the target population. The available data on the prevalence of anaemia among children of diverse population groups clearly demonstrate that the extent of the problem is astronomical, and the success in combating childhood anaemia depends on comprehending its associated factors. As a result, the implication of the problem necessitates additional comprehensive strategy for sustainable long-term approaches, along with short-term measures for immediate prevention and control of anaemia.

Our study recommends that the high prevalence of mild and moderate anaemia demands due emphasis so as to tackle the overall prevalence of anaemia among children aged 0-6 year(s). Children should be periodically screened, and appropriate measures should be taken for detection and preclusion.

Active collaboration among the government, donor agencies, local academic institutions, non-governmental organizations, and local communities is idyllic and urgently desired.

## ACKNOWLEDGEMENTS

The authors wish to thank the reviewers for their constructive suggestions which led to improvement in the presentation of the paper.
